# Molecular, microbiological and clinical characterization of *Clostridium difficile* isolates from tertiary care hospitals in Colombia

**DOI:** 10.1371/journal.pone.0184689

**Published:** 2017-09-13

**Authors:** Clara Lina Salazar, Catalina Reyes, Santiago Atehortua, Patricia Sierra, Margarita María Correa, Daniel Paredes-Sabja, Emma Best, Warren N. Fawley, Mark Wilcox, Ángel González

**Affiliations:** 1 Research Group in Anaerobic Bacteria (GIBA), School of Microbiology, Universidad de Antioquia, Medellín, Colombia; 2 Basic and Applied Microbiology Research Group (MICROBA), School of Microbiology, Universidad de Antioquia, Medellín, Colombia; 3 San Vicente Fundación University Hospital, Medellín, Colombia; 4 Clínica León XIII, IPS Universitaria, Universidad de Antioquia, Medellín, Colombia; 5 Molecular Microbiology Group, School of Microbiology, Universidad de Antioquia, Medellín, Colombia; 6 Microbiota-Host Interactions and Clostridia Research Group, Departamento de Ciencias Biológicas, at Universidad Andres Bello, Santiago, Chile; 7 Departament of Microbiology, Leeds Teaching Hospitals NHS Trust, Leeds, United Kingdom; University of Arizona, UNITED STATES

## Abstract

In Colombia, the epidemiology and circulating genotypes of *Clostridium difficile* have not yet been described. Therefore, we molecularly characterized clinical isolates of *C*.*difficile* from patients with suspicion of *C*.*difficile* infection (CDI) in three tertiary care hospitals. *C*.*difficile* was isolated from stool samples by culture, the presence of A/B toxins were detected by enzyme immunoassay, cytotoxicity was tested by cell culture and the antimicrobial susceptibility determined. After DNA extraction, *tcd*A, *tcd*B and binary toxin (*CDT*a/*CDT*b) genes were detected by PCR, and PCR-ribotyping performed. From a total of 913 stool samples collected during 2013–2014, 775 were included in the study. The frequency of A/B toxins-positive samples was 9.7% (75/775). A total of 143 isolates of *C*.*difficile* were recovered from culture, 110 (76.9%) produced cytotoxic effect in cell culture, 100 (69.9%) were *tcd*A+/*tcd*B+, 11 (7.7%) *tcd*A-/*tcd*B+, 32 (22.4%) *tcd*A-/*tcd*B- and 25 (17.5%) *CDT*a+/*CDT*b+. From 37 ribotypes identified, ribotypes 591 (20%), 106 (9%) and 002 (7.9%) were the most prevalent; only one isolate corresponded to ribotype 027, four to ribotype 078 and four were new ribotypes (794,795, 804,805). All isolates were susceptible to vancomycin and metronidazole, while 85% and 7.7% were resistant to clindamycin and moxifloxacin, respectively. By multivariate analysis, significant risk factors associated to CDI were, staying in orthopedic service, exposure to third-generation cephalosporins and staying in an ICU before CDI symptoms; moreover, steroids showed to be a protector factor. These results revealed new *C*. *difficile* ribotypes and a high diversity profile circulating in Colombia different from those reported in America and European countries.

## Introduction

*Clostridium difficile*, a common causal agent of diarrhea in hospitalized patients, is associated with substantial morbidity and mortality [1]. The clinical manifestations of *C*. *difficile* infection (CDI) range from mild, self-limiting diarrhea to fulminant colitis that include pseudomembranous colitis and toxic megacolon [2]. CDI is a hospital-acquired infection; but lately, *C*. *difficile* has been -associated with diarrhea in the community [2]. Several antibiotic classes have been associated with the development of CDI, including clindamycin, cephalosporins and fluoroquinolones [3,4]. Oral metronidazole is usually used to treat mild to moderate CDI, and vancomycin, severe infection [5]; although, resistance to vancomycin is not yet a major issue, reduced susceptibility to this antibiotic has been reported [6–8].

*Clostridium difficile* produces two major toxins, toxin A and toxin B that are encoded by the *tcdA* and *tcdB* genes, respectively, and are responsible for the pathogenicity of this bacterium. In addition, some isolates produce a binary toxin, which has been associated with the severity of the disease [9,10]. Various laboratory tests are available to diagnose CDI, they include isolation of toxigenic *C*. *difficile* by culture, detection of A and B toxins by immunoassays, and molecular assays to detect toxin genes in stool samples [11–13].

PCR ribotyping is a molecular tool employed for the global analysis of *C*. *difficile* related virulent strains based on a reference library. Thus, various ribotypes have been described in Europe and United States; nonetheless, the most prevalent ribotypes are 027, 001/072 and 014/020 [1]. Particularly, in recent years, the virulent or epidemic strain NAP1/PCR ribotype 027 and to a lasser extent ribotype 078 have been associated with an increase on the incidence and mortality rates in North America and European countries [2]. In Latin America, some CDI cases have documented and the NAP1/027 ribotype was reported in Costa Rica, Panama, México, Chile and Colombia [14–19]. Other ribotypes including 014, 106, 010, 020, 133 and 233 have been registered in Brazil, but 027/078 have not yet been detected [20–22]. Although, ribotye NAP1/027 has been reported in Colombia [23], the diversity of circulating genotypes remains to be described. Therefore, the aim of the present work was to characterize at the molecular and microbiological level *C*. *difficile* isolates, and to analyse the clinical characteristics of patients with CDI from three high compelxity hospitals in Medellín, Colombia.

## Materials and methods

### Patients and study design

A prospective cross-sectional study was conducted during January 2013 to December 2014 in three tertiary care hospitals in Medellín, Colombia. Hospital A had 731 beds, hospital B 650 beds and hospital C 653 beds. All patients with suspicion of CDI and those who received at least one dose of antimicrobial therapy six weeks before the symptoms started, were involved in the study. A CDI case was defined as a patient with clinical suspicion of the disease, and with a stool positive for *C*. *difficile* A/B toxins. CDI was classified as mild, moderate, severe or complicated according to guidelines by the American College of Gastroenterology (ACG) [24]. Clinical and demographic data were obtained from medical records, after patients have read and signed an informed consent.

### Ethics statement

This work was approved by the Human Research Ethics Committee of Universidad de Antioquia (Comité de Bioética, Sede de Investigación Universitaria, CBEIH- SIU, approval number 12-35-458).

### Toxin detection, *C*. *difficile* culture and identification on stool samples

Samples were processed immediately after arrival at the Lab or refrigerated for no more than 72 h before being processed. Stool samples were initially tested for free *C*. *difficle* A/B toxins using one or two enzyme immunoassays (EI) ImmunoCard toxin A&B (Meridian Bioscience, Cincinnati, OH) or MiniVidas *C*. *difficile* Toxin A/B assay (bioMerieux, Marcy I´Etoile, France). All samples were inoculated on cycloserine-cefoxitin-fructose-taurocholate agar (TCCFA) [25], and incubated at 37°C for 48 h in an anaerobic chamber (90% N_2_, 5%CO_2_, 5%H_2_). From *C*. *difficile* positive TCCFA, a single colony was subcultured on blood agar and then identified on the basis of colony morphology, fluorescence under UV light, L-proline aminopeptidase production and confirmed using the API Rapid ID 32A system (BioMérieux Inc., Durhan, NC, USA). Isolates not clearly confirmed as *C*. *difficile* were subjected to matrix- assisted laser desorption ionization–time of flight mass spectrometry (MALDI-TOF MS). *C*. *difficile* isolates were stored at -70°C in brain-heart infusion (BHI) broth (Becton Dickinson, Franklin Lakes, NJ, USA) supplemented with 20% glycerol, for further analysis.

### Antimicrobial susceptibility testing

Antimicrobial susceptibility to metronidazole and vancomycin was determined by minimum inhibitory concentration (MIC) using the standard agar dilution method, following guidelines by the Clinical and Laboratory Standards Institute (CLSI) [26], and susceptibility to clindamycin and moxifloxacin, were tested by the epsilometric method (Etest BioMérieux, Marcy l’Etoile, France). To perform the agar dilution method, *C*. *dificille* isolates were grown on Brucella agar (Oxoid Ltd., Basingstoke, UK) previously supplemented with 5 μg hemin, 1 μg Vitamin K1 per mL and 5% v/v laked blood sheep, and mixed with the antimicrobial agent solution; for the case of the epsilometric method, we used the same Brucella agar but instead of laked we used defibrinated blood sheep. Susceptible or resistant microorganisms used as controls were, *B*. *fragilis* ATCC 25285 and *C*. *difficile* ATCC 700057.

### Cytotoxicity assay

For cytotoxicity assays, a colony of *C*. *difficile* was inoculated into BHI broth and incubated under anaerobic conditions for 48 h at 37°C. After centrifugation, 100 μl of each culture supernatant was added to a confluent monolayer of Vero cells in 96-well plate containing Dulbecco’s Modified Eagle’s Medium (DMEM, HyClone, Thermo Scientific), supplemented with 10% fetal bovine serum (FBS, HyClone), for a final concentration of 5x10^3^ cells/well. Cells were cultured at 37°C, 5% CO_2_ and examined to evaluate the cytopathic effect at 24 and 48 hours. Positivity was confirmed by neutralization using a polyclonal goat antiserum anti-*C*. *difficile* toxins A/B (TechLab^®^, Blacksburg, VA) [27].

### Detection of *C*. *difficile* toxin genes

DNA was extracted from cultures grown in BHI broth, incubated overnight in anaerobic conditions, using the DNA isolation kit (MoBio Laboratories Inc., Carlsbad, CA) and following the manufacturer instructions. The *tcd*A (toxin A) and *tcd*B (toxin B) genes were determined using the NK2/NK3 and NK104/NK105 primers respectively, to amplify a 252-bp fragment for *tcd*A and 203-bp fragment for *tcdB* [28]. The binary toxins genes were detected using the *cdt*Apos/*cdt*Arev and *cdt*Bpos/*cdt*Brev primers that amplify a 375-bp fragment for the *cdtA* gene and 510-bp fragment for *cdtB*, and following previously described protocols [29,30]. Conditions for amplification were: 35 cycles of denaturation at 95°C for 45s (*tcdA* and *tcdB*) and (*cdtA* and *cdtB*), annealing at 58.4°C for 60s (*tcdA*), 60°C for 60s (*tcdB*), 63.9°C for 60s (*cdtA*) and 56.4°C for 60s (*cdtB*) and extension at 72°C for 60s. PCR products were visualized in 1.5% to 2% agarose gels stained with ethidium bromide.

### PCR ribotyping

The PCR ribotyping was performed using a high-resolution capillary gel-based electrophoresis after amplification of the 16S–23S intergenic spacer, following protocols of Department of Microbiology, Leeds Teaching Hospitals NHS Trust, Leeds, UK [31]. Briefly, PCR products were analyzed using an ABI-PRISM 313xl automated sequencer and fragment analysis system, a 16 capillary 36 cm array with POP-7 separation matrix (Life Technologies, Paisley, UK) and a GeneScan 600 LIZ as an internal marker. Bio- Numerics v.7.1 software (Applied Maths, Sint-Martens-Latem, Belgium) was used to import the fluorescent signals and GeneMapper v.4.0 software (Applied Biosystems, Life Technologies, Grand Island, NY) was used to size the fragments [31]. Cluster analysis of PCR ribotype band profiles was performed using the DICE similarity coefficient with relationships represented in a UPGMA dendrogram within Bio-Numerics v.7.1 software. Ribotypes were identified by comparison of the band profiles with those in the reference UK library (*Clostridium difficile* Network for England and Northern Ireland).

### Statistical analysis

Questionnaire data were stored in a Microsoft Access database. Statistical analysis was performed using the SPSS v. 23.0 statistic software package (IBM-SPSS Inc, Armonk, NY) and Stata v.13.0 software (StataCorp LP, College Station, TX). Data were described using absolute and relative frequencies, and media and standard deviation for categorical and numerical variables, respectively. Group comparisons for categorical and numerical variable types were performed using chi-square and Mann Whitney U tests, respectively. Associations between clinical variables and CDI were analyzed using multivariate logistic regression analysis; variables with a *P*<0.25 in the bivariate analysis were considered in the logistic stepwise regression performed with the Hosmer-Lemeshow criteria, to find the best model that fit the data with the lowest Bayesian information criterion (BIC), and those with *P* < 0.05 were considered significant in the final model.

## Results

### Frequency of CDI according to positive free toxin in stool samples

A total of 913 stool samples from patients with suspicion of CDI were analyzed. One hundred and thirty-eight (15.1%) samples were excluded because they did not meet the antibiotics usage critera or were duplicated. From a total of 775 samples analyzed, 487 (62.9%) were from hospital A, 190 (24.5%) from hospital B and 98 (12.6%) from hospital C. The global frequency of free positive toxin in stool samples was 9.7% (75/775). The frequency of CDI in each hospital was 7.0%, 17.9% and 7.1% for hospitals A, B and C, respectively.

### Demographic and clinical characteristics of patients with suspicion of CDI according to positive stool toxin

Several clinical characteristics and laboratory data from patients with (CDI) or without (non-CDI) stool toxin were compared in a bivariade analysis (Tables 1 and 2). The median age of CDI patients involved in this study was 63 years with an interquartile range (IQR) from 47 to 78, and 49.3% were female. Thirty-three patients (44%) with CDI were older than 65 years. There was not significant difference in sex or age betwen CDI and non-CDI individuals. The median of length of stay (LOS) at the hospital was 27 days (IQR 15–44 days) for CDI patients versus 25 days (IQR 13–41) for those non-CDI individuals. Of note, a significant difference was observed between CDI patients staying in ICU before presenting the symptoms as compared to those negative for the *C*. *difficile* toxin in stools (29.3% versus 15%, *P* = 0.001).

**Table 1:** Demographic and clinical characteristics of those patients with and without stool toxin.

Variable	*C*. *difficile* toxin positive (*n* = 75) *n* (%)	*C*. *difficile* toxin negative (*n* = 700) *n* (%)	*P* value
**Age (years)**			
Median	63	65	>0.05*
Interquartile range	47–78	48–77	
Older 65 years	33 (44)	340 (48.5)	0.451
Female	37 (49.3)	390 (55.7)	0.291
**Comorbidities**			
Coronary disease	40 (53.3)	363 (51.8)	0.817
Kidney disease	25 (33.3)	226 (32.2)	0.854
Sepsis	17 (22.7)	154 (22.0)	0.895
Diabetes	17 (22.7)	174 (24.8)	0.676
Thyroid disorder	9 (12)	82 (11.7)	0.894
**System or organ affected**			
Musculoskeletal	16 (21.3)	66 (9.4)	0.001
Urinary	15 (20)	122 (17.4)	0.579
Digestive/Intestinal	13 (17.3)	180 (25.7)	0.111
Central Nervous	10 (13.3)	69 (9.8)	0.344
Respiratory	9 (12)	146 (20.9)	0.068
Cardiovascular	6 (8)	52 (7.4)	0.858
Hematological / Immune	5 (6.7)	96 (13.7)	0.085
Genital	4 (5.3)	5 (0.7)	<0.001
Orthopedic service	14 (18.7)	39 (5.6)	<0.001
**Risk factors**			
Proton pump inhibitors	64 (85.3)	552 (78.9)	0.187
Previous hospitalization	39 (52)	310 (44.3)	0.202
Infection during hospitalization	41 (54.7)	353 (50.4)	0.485
Tube nasogastric	17 (22.7)	124 (17.7)	0.291
Steroids	15 (20)	250 (35.7)	0.006
Dialysis	13 (17.3)	99 (14.1)	0.455
Endoscopy	12 (16)	112 (16)	1.000
Abdominal surgery	10 (13.3)	106 (15.1)	0.676
Enema	3 (4)	58 (8.3)	0.229
Colonoscopy	2 (2.7)	49 (7.0)	0.178
LOS (days)			
Median	27	25	0.202
Interquartile range	15–44	13–41	
Stay ICU before CDI Symptoms	22 (29.3)	105 (15)	0.001
**Discharge**			
Death	15 (20)	97 (13.9)	0.150
Improved symptoms	59 (78.7)	584 (83.4)	0.297
**Previous usage of antibiotics**			
Penicillins			
Penicillin G	6 (8.0)	42 (6.0)	0.495
Piperacillin/tazobactam	37 (49.3)	403 (57.6)	0.171
Ampicillin/sulbactam	13 (17.3)	137 (19.6)	0.641
Metronidazole	12 (16.0)	123 (17.6)	0.733
Carbapenem (Meropenem)	31 (41.3)	214 (30.6)	0.512
Cephalosporin			
1st Generation	8 (10.7)	59 (8.4)	0.057
3rd generation	16 (21.3)	51 (7.3)	<0.001
4th generation	8 (10.7)	53 (7.6)	0.344
Glycopeptides (vancomycin)	21 (28)	169 (24.1)	0.461
Aminoglycosides	9 (12.0)	74 (10.6)	0.704
Macrolides (Clarithromycin)	5 (6.6)	106 (15.1)	0.046
Lincosamides (Clindamycin)	8 (10.7)	60 (8.6)	0.542
Fluoroquinolones (Ciprofloxacin)	15 (20)	148 (21.1)	0.817
Oxazolidinones (Linezolid)	6 (8.0)	85 (12.1)	0.290

CDI, *C*. *difficile* infection; LOS, length of stay;

* Mann-Whitney test

**Table 2:** Laboratory data, clinical signs and symptoms of those patients with and without stool toxin.

Laboratory data, clinical signs/symptoms	*C*. *difficile* toxin positive *n* (%)	*C*. *difficile* toxin negative *n* (%)	*P* value
White Blood Cell counts			
Median	12100	8740	<0.001
Interquartile range	7900–19900	6260–12425	
C-Reactive Protein (CRP)			
Median	9.44	6.6	0.060
Interquartile range	3.40–16.88	2.68–13.9	
Blood in stool	3 (4.0)	13 (1.9)	0.215
Ileus	4 (5.3)	6 (0.9)	0.005
Hypotension	8 (10.7)	37 (5.3)	0.159
Fever	13 (17.3)	81 (11.6)	0.146
Abdominal pain	33 (44.0)	176 (25.1)	<0.001
Abdominal distention	16 (21.3)	107 (15.3)	0.173
Nausea	7 (9.3)	16 (2.3)	0.001
Vomit	8 (10.7)	34 (4.9)	0.035
Diarrhea	70 (93.3)	644 (92.0)	0.684
Septic Shock	2 (2.7)	11 (1.6)	0.483

CDI, *C*. *difficile* infection

Furthermore, analyses of comorbidities, systems or organs affected, risk factors, death or improvement of the CDI patients as well as previous administration of antibiotics (Table 1), showed significant differences on those CDI patients who have affected the musculoskeletal (21.3% versus 9.4%) and genital (5.3% versus 0.7%) systems or those who assisted to orthopedic service (18.7% versus 5.6%), as compared to those non-CDI patients. Among the risk factors analyzed, only steroid usage showed a significant difference between CDI and non-CDI patients (20% versus 35.7%, *P* = 0.006).

The most frequently administered antibiotics during the six week period prior symptoms or toxin test were, piperacillin/tazobactam administered to 440 (56.7%) patients, followed by meropenem: 245 (31.6%), vancomycin: 190 (24.5%), ciprofloxacin: 163 (21.0%), ampicillin/sulbactam: 150 (19.3%), metronidazole: 135 (17.4%), clarithromycin: 111 (14.3%), linezolid: 91 (11.7%) and others, at less than 10% each. Among the different antibiotics used, only third generation cephalosporin and macrolides showed a significant difference between CDI and non-CDI patients (Table 1).

Regarding the severity of the diseases, 58 patients had mild-moderate CDI; from these 30 (51.7%) patients received only oral metronidazole and 6 (10.3%) received both oral metronidazole and vancomycin. Six patients had severe disease and two severe complicated, and nine patients were not classified. A total of 69 (92%) of CDI patients recieved therapy for this disease, while five patients were discharged and one died before starting the specific treatment. About the laboratory data and clinical signs and symptoms, CDI patients showed significant differences in white blood cell counts, ileus, abdominal pain, nausea and vomit, in comparison with those patients with negative *C*. *difficile* toxin (Table 2).

In addition, 51% of the patients (394/775) had a concomitant infection; of these, 143 (36.3%) had urinary tract and 97 (24.6%) bloodstream infections. The most frequent pathogens isolated from patients with these infections were *Escherichia coli* and *Staphylococcus aureus*, respectively (data not shown). Moreover, from 644 patients with diarrhea, 8 (1.2%) had other pathogens as possible cause of diarrhea; one of them had *Salmonella enterica*, two *Strongyloides stercolaris* and 5 *Entamoeba histolytica*.

The bivariate analysis to compare CDI versus non-CDI patients conduced to identify variables associated with the development of CDI, showed that staying in an orthopedic service (OR 3.8, 95% CI 2.0–7.56) or ICU (OR 3.45, 95% CI 1.8–6.42), administration of third-generation cephalosporins (OR 1.98, 95% CI 1.2–3.2), and affection of the musculoskeletal system (OR 2.6, 95% CI 1.41–4.78) before CDI symptoms, were factors associated with CDI. Interestingly, steroid usage was associated as a protector factor (OR 0.45, 95% CI 025–080) (Table 3).

**Table 3:** Results of bivariate analysis to compare CDI versus those non-CDI patients.

	Odds Ratio	CI 95%	*P* value
Age > 65	0.83	0.51–1.34	0.452
> 3 antibiotics	1.25	0.77–2.01	0.355
Carbapenem	1.6	0.98–2.6	0.058
3rd generation cephalosporin	3.45	1.8–6.42	0.000
Glycopeptides	1.2	0.71–2.06	0.47
Macrolides	0.40	0.15–1.01	0.054
Fluoroquinolones	0.93	0.51–1.68	0.817
Oxazolidinones (linezolid)	0.62	0.26–1.49	0.293
Piperacillin/Tazobactam	0.71	0.44–1.15	0.173
Proton pump inhibitors	1.55	0.80–3	0.190
Steroids	0.45	0.25–080	0.008
Nasogastric tube	1.36	0.76–2.41	0.26
Abdominal surgery	0.86	0.42–1.73	0.677
Stay IUC before Symptoms	1.96	1.2–3.2	0.007
Orthopedic Service	3.8	2.0–7.56	0.001
Musculoskeletal Systems	2.6	1.41–4.78	0.002
Previous hospitalization	1.36	0.84–2.19	0.203

CDI, *C*. *difficile* infection; ICU, Intensive Care Unit.

A multivariate logistic regresision model was performed using a stepwise selection including variables that showed significant outcomes or a *P* value <0.25 in the bivariate analysis; this analysis showed that staying in an orthopedic service (OR 3.97, 95% CI 1.98–7.93), exposure to third-generation cephalosporins (OR 3.91, 95% CI 2.06–7.46), staying in ICU before CDI symptoms (OR 2.01, 95% CI 1.20–3.36), remained as significant risk factors associated with CDI, and as described above, steroid usage (OR 0.45, 95% CI 0.22–0.75) appears to be a protective factor for CDI (Table 4).

**Table 4:** Multivariate analysis of *Clostridium difficile* infection risk factors.

Factor	Adjusted Odds Ratio	CI 95%	*P* value
Third-generation cephalosporin	3.91	2.06–7.46	0.000
Stay in ICU before CDI symptoms	2.01	1.20–3.36	0.000
Steroids	0.45	0.22–0.75	0.004
Orthopedic Service	3.97	1.98 7.93	0.000

### Detection of *C*. *difficile* toxin genes

A total of 143 *C*. *difficile* isolates were obtained by culture. From these, 71 corresponded to patients with positive *C*. *difficile* toxin, and from these, 62 (87.3%) harbored the *tcdA*/*tcd*B genes, six (8.5%) were *tcdA*-/*tcd*B+ and 3 (4.2%) were negative for these toxin genes (Table 5). The remaining 72 *C*. *difficile* isolates corresponded to non-CDI patients. From these 72 isolates, 39 (54.2%) were *tcdA*+/*tcd*B+ and four (5.6%) *tcdA*-/*tcd*B+ and 29 (40.2%) were negative for these genes. Only 25 isolates were positive for the binary toxin (*CDT*a/*CDT*b) (Table 5); from these, 20 (80%) were from patients with CDI and five (20%) from non-CDI patients (data not shown).

**Table 5:** Distribution of toxin genes profiles, toxin EIA and cytotoxicity in culture of *C*. *difficile* isolates (n = 143).

Toxin genes	Toxin EIA		Binary toxin *CDTa*/*CDTb*		Cytotoxicity	
	Positive	Negative	Positive	Negative	Positive	Negative
*tcdA*+ / *tcdB*+	62	39	25	0	100	1
*tcdA*- / *tcdB*+	6	4	0	0	10	0
*tcdA*+ / *tcdB*-	0	0	0	0	0	0
*tcdA*- / *tcdB*-	3	29	0	0	0	32

EIA, Enzyme immunoassay

### *Clostridium difficile* clinical isolates induce a cytotoxic effect on Vero cells

In the cytotoxicity assays, 110 from the 143 *C*. *difficile* isolates produced a cytotoxic effect in cell culture; from these, 100 (90.1%) were *tcdA*+/*tcd*B+ and 10 (9.9%) *tcdA*-/*tcd*B+. The remaining 33 isolates did not show cytotoxic effect, and 32 (97%) of them were negative for toxin genes, and only one (3%) harbored these genes (*tcdA*+/*tcd*B+) (Table 5). In addition, from the 110 cytotoxic isolates, 67 (60.9%) were from CDI patients, while 43 (39.1%) were from non-CDI patients. From the 33 isolates with a negative cytotoxic effect, 29 (87.9%) were from non-CDI and four (12.1%) from CDI patients (data not shown).

### Diversity of *C*. *difficile* PCR ribotypes

**Fig 1:**
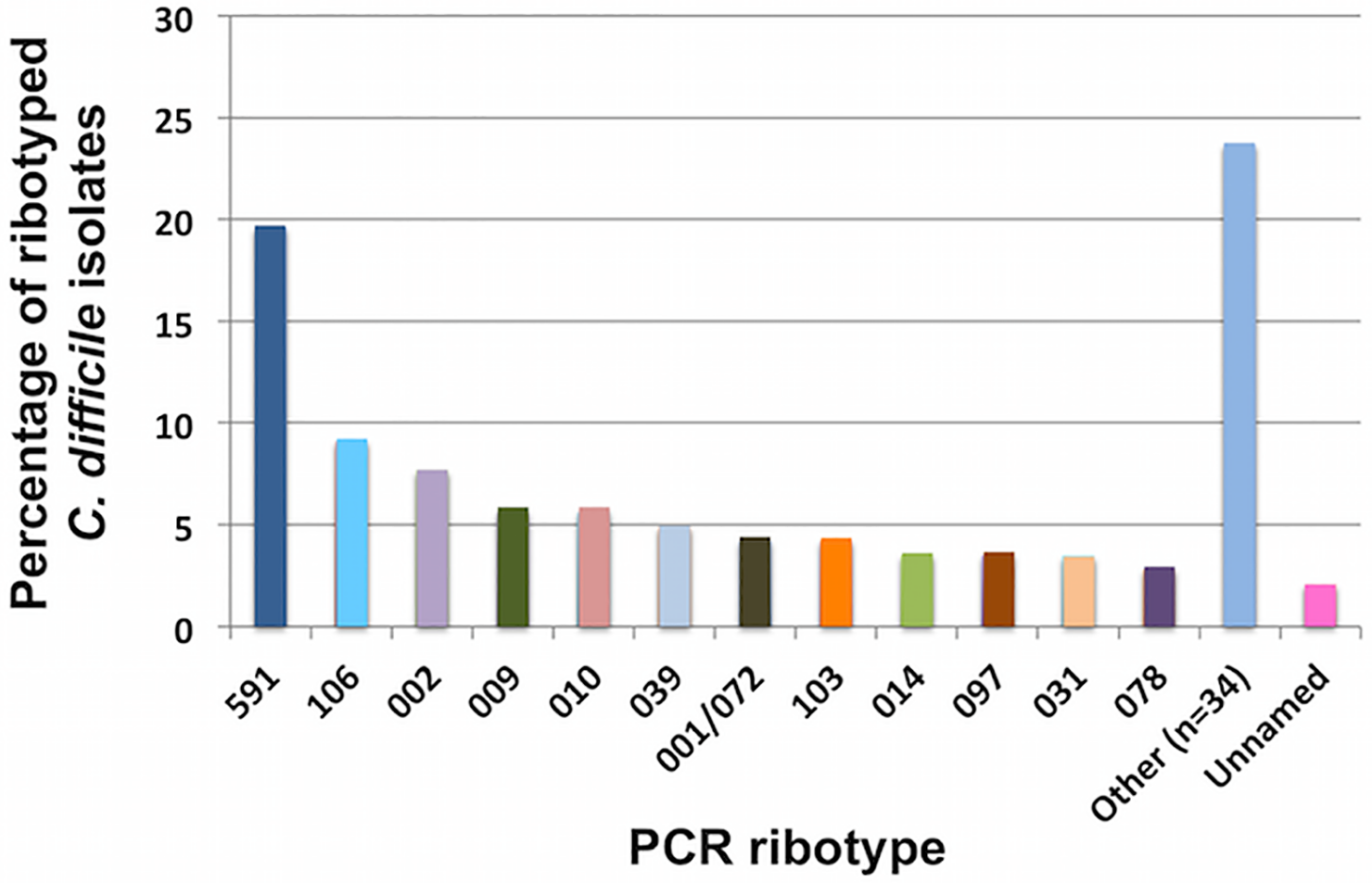
Distribution of the most commonly isolated *C*.*difficile* PCR Ribotypes from all *C*. *difficile* islates in three tertiary care hosiptals in Medellín, Colombia, 2013–2014 (*n* = 143). Other column includes the ribotypes 005, 012, 015, 027, 046, 050, 054, 056, 057, 075, 076, 137, 138, 151, 173, 194, 255, 287, 353, 354, 451, 580, 794, 795, 804/805.

A total of 37 *C*. *difficile* ribotypes were identified, 3 isolates produced unidentified ribotypes (unnamed). Among the 12 most frequent ribotypes (Fig 1), ribotype 591 was the most prevalent (19.6%, *n* = 28), followed by ribotypes 106 (13%, *n* = 13), 002 (7.7%, *n* = 11), 009 (5.6%, *n* = 8) and 010 (5.6%, *n* = 8). Remarkably only four isolates corresponded to ribotype 078 (2.8%) and one to ribotype 027 (0.7%). Further, four new ribotypes were detected, named 794, 795, 804 and 805.

**Fig 2:**
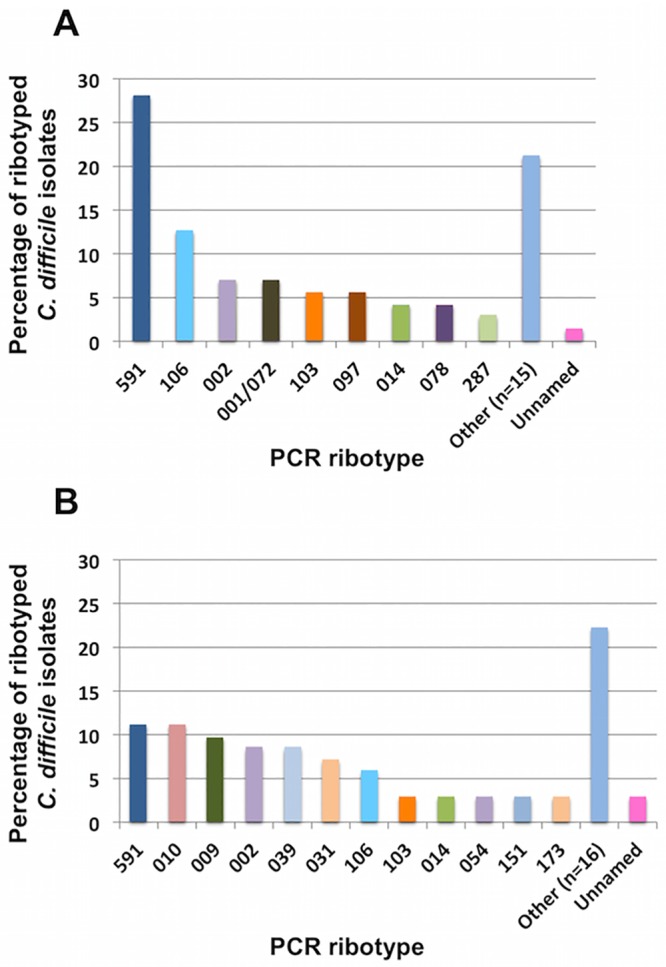
Distribution of the most commonly isolated *C*.*difficile* PCR Ribotypes from (A) cases of CDI (71 isolates) and (B) patients with negative *C*. *difficile* toxin (72 isolates) in three tertiary care hosiptals in Medellín, Colombia, 2013–2014 (*n* = 143). Other column for CDI cases includes the ribotypes 005, 009, 010, 012, 015, 027, 031,039, 046, 050, 054, 057, 056, 075, 076, 137, 138, 151, 173, 194, 255, 353, 354, 451, 580, 794, 795, 804/805; while other column for negative *C*. *difficile* toxin includes the ribotypes 001/072, 005, 012, 015, 027, 046, 050, 056, 057, 075, 076, 078, 097, 137, 138, 194, 255, 287, 353, 354, 451, 580, 794, 795, 804/805.

The most prevalent ribotypes from CDI patients were, 591 (28%, *n* = 20), 106 (12.6%, *n* = 9), 002 (7%, *n* = 5), 001/072 (7%, *n* = 5), 103 (5.6%, *n* = 4) and 097 (5.6%, *n* = 4) (Fig 2A), while those from non-CDI patients were, 591 (11.1%, *n* = 8), 010 (11.1%, *n* = 8), 009 (9.7%, *n* = 7), 002 (8.3%, *n* = 6), 039 (8.3%, *n* = 6) and 031 (6.9%, *n* = 5) (Fig 2B).

A total of 68 isolates were from hospital A, 57 from hospital B and 18 from hospital C (Table 6). The most prevalent ribotypes in hospital A were 591 (20.5%, *n* = 14) and 002 (11.7%, *n* = 8), 591 (21%, *n* = 12) and 106 (12.3%, *n* = 7) in hospital B, and 001/072 (22.2%, *n* = 4) and 591 (11.1%, *n* = 2) in hospital C.

**Table 6:** Frequency of PCR ribotypes among *C*. *difficile* isolates from the different tertiary care hospitals in Medellín, Colombia, 2011–2012 (n = 143).

PCR ribotype	Hospital A No. isolates (%)	Hospital B No. isolates (%)	Hospital C No. isolates (%)	Total No. isolates (%)
591	14 (20.5%)	12 (21.0%)	2 (11.1%)	28 (19,6)
106	6 (8.8%)	7 (12.3%)	0 (0%)	13 (9,1)
002	8 (11.7%)	2 (3.5%)	1 (5.6%)	11 (7,7)
009	5 (7.4%)	3 (5.2%)	0 (0%)	8 (5,6)
010	6 (8.8%)	1 (1.7%)	1 (5.6%)	8 (5,6)
039	5 (7.4%)	2 (3.5%)	0 (0%)	7 (4,9)
001/072	0 (0%)	2 (3.5%)	4 (22.2%)	6 (4,2)
103	4 (5.9%)	2 (3.5%)	0 (0%)	6 (4,2)
014	1 (1.5%)	3 (5.2%)	1 (5.6%)	5 (3,5)
097	1 (1.5%)	4 (7.0%)	0 (0%)	5 (3,5)
031	4 (5.9%)	0 (0%)	1 (5.6%)	5 (3,5)
078	3 (4.4%)	0 (0%)	1 (5.6%)	4 (2,8)
054	1 (1.5%)	2 (3.5%)	0 (0%)	3 (2,1)
173	0 (0%)	3 (5.2%)	0 (0%)	3 (2,1)
050	0 (0%)	2 (3.5%)	0 (0%)	2 (1,4)
353	0 (0%)	2 (3.5%)	0 (0%)	2 (1,4)
354	2 (2.9%)	0 (0%)	0 (0%)	2 (1,4)
151	0 (0%)	1 (1.7%)	1 (5.6%)	2 (1,4)
287	2 (2.9%)	0 (0%)	0 (0%)	2 (1,4)
005	0 (0%)	1 (1.7%)	0 (0%)	1 (0,7)
012	1 (1.5%)	0 (0%)	0 (0%)	1 (0,7)
015	0 (0%)	0 (0%)	1 (5.6%)	1 (0,7)
027	0 (0%)	0 (0%)	1 (5.6%)	1 (0,7)
046	0 (0%)	0 (0%)	1 (5.6%)	1 (0,7)
057	0 (0%)	1 (1.7%)	0 (0%)	1 (0,7)
076	1 (1.5%)	0 (0%)	0 (0%)	1 (0,7)
137	1 (1.5%)	0 (0%)	0 (0%)	1 (0,7)
138	0 (0%)	0 (0%)	1 (5.6%)	1 (0,7)
194	0 (0%)	1 (1.7%)	0 (0%)	1 (0,7)
255	1 (1.5%)	0 (0%)	0 (0%)	1 (0,7)
451	0 (0%)	1 (1.7%)	0 (0%)	1 (0,7)
580	1 (1.5%)	0 (0%)	0 (0%)	1 (0,7)
794	0 (0%)	1 (1.7%)	0 (0%)	1 (0,7)
795	1 (1.5%)	0 (0%)	0 (0%)	1 (0,7)
056	0 (0%)	1 (1.7%)	0 (0%)	1 (0,7)
075	0 (0%)	0 (0%)	1 (5.6%)	1 (0,7)
804/805	0 (0%)	1 (1.7%)	0 (0%)	1 (0,7)
Unnamed	0 (0%)	2 (3.5%)	1 (5.6%)	3 (2.1)
Total	68 (100%)	57 (100%)	18 (100%)	143 (100)

### Antibiotic susceptibility of *C*. *difficle* isolates

In the antimicrobial susceptibility assays, the 143 isolates (100%) were susceptible to vancomycin and metronidazole, while 11 (7.7%) and 126 (86.1%) isolates were resistant to moxifloxacin and clindamycin, respectively. The minimum inhibitory concentrations (MIC50 and MIC90) for vancomycin, metronidazole, moxifloxacin and clindamycin were 0.5–1.0 μg/mL, 0.25–0.5 μg/mL, 1.5–2.0 μg/mL and 8.0–256 g/mL, respectively. Breakpoints to characterize isolates as susceptible or resistant were as follow: metronidazole (susceptible ≤8 μg/mL, resistant ≥32 μg/mL), clindamycin, moxifloxacin and vancomycin (susceptible ≤2 μg/mL, resistant ≥8 μg/mL). Intermediate breakpoints were considered as resistant. Notoriously, all isolates (100%) of the most prevalent ribotype 591, were resistant to clindamycin, while only four (14.3%) were resistant to moxifloxacin. Interstinlgy, the only isolate detected of ribotype 027 was susceptible to all of the antibiotics tested (data not shown).

## Discussion

Little is known about the epidemiology of CDI in Colombia. Therefore, in this study a cohort of patients with suspicion of CDI was evaluated to report for the first time the diversity of circulating ribotypes, and new ribotypes are described.

Most of the risk factors frequently reported in several studies for the development CDI include, number and type of antibiotics, patients older than 65 years [32,33], length of stay (LOS) at the hospital [34,35], nasogastric tube insertion [36], and various comorbidities or preexisting conditions [23,34,37,38]; nonetheless, in this study, these variables did not show significant associations.

The only two studies previously conducted in Colombia reported as CDI associated factors, age over 65 years, proton pump inhibitors (PPIs) usage, previous administration of third-generation cephalosporins and staying in ICU before CDI symptoms; and the comorbidities associated with CDI were diabetes mellitus and leukaemia [23]. In concurrence with these studies, in the present study the multivariate regression model performed indicated that staying in ICU before CDI symptoms, previous administration of third-generation cephalosporins and staying in an orthopedic service were associated factors. For instance, patients at the orthopedic service, subjected to surgery and to extend antibiotic therapies, are most likely to develop CDI; also steroids were a protector factor. Similarly, a previous study conducted in a geriatric hospital as well reported that steroids were more frequently used in patients without CDI in comparison with those eldery patients who developed CDI [37]. These results contrast with those of investigations that indicate that steroids are potentially useful markers for CDI mortality prediction [38,39]. Moreover, in a mouse model it was demostrated that immunosuppressive drugs, such steroids, increase the severity of CDI by alteration of the gut microbiota [40]. These controversial results indicate the need of conducting additional studies to determine the effect of steroids on the gut microbiota and inflammatory response influencing the severity of CDI.

Also in this study and in agreement with various reports, no significant association of CDI development with PPIs or histaminic receptor-blocking gastric acid suppressors was observed; however, other studies have shown contrasting results indicating an association of PPIs with a decreased risk for CDI development in those who received antibiotics [41–43].

Several antibiotics have been associated with CDI development in hospitals, these include cephalosporins, clindamycin, penicillins, metronidazole, vancomycin and fluoroquinolones [3,4]. From these, cephalosporins are the most frequently antibiotics implicated in CDI because of their widely effect on gut microbiota [10]. Noteworthy, in this study, cephalosporins were administered to 25% of the patients. First-generation cephalosporins were regularly used as the prophylactic treatment previous to a surgery. An association was found between third-generation cephalosporins and CDI, similar to previously reported [3,4].

In the present study the frequency of CDI cases was 9.7%; this frequency is lower than the one observed in others studies [1,44]. Thus, a total of 75 from 775 patients were classified as CDI; nonetheles, 143 isolates were obtained by culture. From these isolates, 71 were from CDI and 72 from non-CDI patients. From the 71 CDI isolates, 68 (95.7%) harbored the *tcd*B+ and 3 (4.3%) were negative for the toxin genes. From the 72 isolates of non-CDI patients, 43 (59.7%) harbored the *tcd*B+ and 29 (40.3%) were negative for the toxin genes. Interestingly, from the 111 isolates harboring the *tcd*B+ genes (68 from CDI and 43 from non-CDI cases), 110 (99.1%) produced toxin in culture; this suggests that perhaps some patients classified as non-CDI were realy CDI cases. Other studies have reported that toxin detection alone or in combination with other methods show a sensitivity that ranges from 67.3% to 84.3% [45,46]. Our results clearly indicate that the combination of two or more diagnostic methods should be implemented in an diagnostic algortim in order to improve CDI diagnosis [33]. In addition, degradation of the toxin in the stool samples during the preanalytic phase due to a delayed processes or samples arriving later to the laboratory could not be ruled out; therefore, other factors could also be considered, i.e. the proportion of false negative microbiological test which may reach 14% after 1 day, and up to 45% after 3 days of processing the clinical samples, independently of the detection method used [47].

The diversity of *C*. *difficile* is commonly evaluated in countries of Europe and United States. Although epidemiology of this infection is changing, the ribotype 027 continues as one of the most prevalent and increasing ribotype in these localities [1,6]. In addition ribotypes such as 001/072, 014/020, 106 and 053 are gaining importance [1,6]. In this work, 37 different ribotypes were decteted, ribotype 591 being the most prevalent, followed by 106, 002, 009 and 010. Of note, only one isolate corresponded to the ribotype 027, four to ribotype 078 and four new ribotypes were reported, named 794, 795, 804 and 805. Interestingly, the most prevalent ribotype, 591, is found at low frequency in Europe and North America, while the ribotypes 106 and 002 were considered epidemic in the United Kingdom during the last decade [48]. Although associations between ribotypes and clinical characteristics or risk factors were not observed, ribotype distribution between CDI and non-CDI patients varied. The most prevalent ribotypes among CDI patients were 591, (28%), 106 (12.6%), 002 (7%), 001/072 (7%), 103 (5.6%) and 097 (5.6%), while non-CDI patients showed ribotypes 591 (11.1%), 010 (11.1%), 009 (9.7%), 002 (8.3%), 039 (8.3%) and 031 (6.9%). These results showed that ribotypes 010 and 031 were only found in non-CDI patients while ribotype 001/072 only in CDI. Of note, ribotype 010 has been reported as non-toxigenic [49], fact that could explain why this ribotype is found only in this group of non-CDI patients. Therefore, further studies should be conducted to stablish associations between CDI and specific ribotypes.

In this study, all *C*. *difficile* isolates were susceptible to metronidazole and vancomycin, including the virulent ribotypes 027 and 001/0072 that in other studies have been associated with resistance to various antimicrobials [2,50]. Of note, 87.7% of the isolates were resistant to clindamycin and 7.7% to moxifloxacin, results that contrast with those from the Pan-European longitudinal surveillance of antibiotic study which reported resistance of 39.9% and 49.6% to moxifloxacin and clindamycin, respectively [50].

## Conclusions

In Colombia this is the first study on *C*. *difficile* ribotype diversity. Results indicate that the epidemiology of CDI, its associated risk factors and ribotype distribution differ with regards to what is reported for the United States and Europe. Finally, the results serve to emphasize the importance of establishing diagnostic algorithms, adapt international guidelines to local conditions and implement a surveillence program to monitor the epidemiology of *C*. *difficile* in Colombia.

## References

[1] Davies KA, Ashwin H, Longshaw CM, Burns DA, Davis GL, Wilcox MH (2016) Diversity of *Clostridium difficil* e PCR ribotypes in Europe: results from the European, multicentre, prospective, biannual, point-prevalence study of *Clostridium difficile* infection in hospitalised patients with diarrhoea (EUCLID), 2012 and 2013. Eur Surveill 29:1–11. 10.2807/1560-7917.ES.2016.21.29.30294 27470194

[2] Smits WK, Lyras D, Lacy DB, Wilcox MH, Kuijper EJ (2016) *Clostridium difficile* infection. Nat Rev Dis Primers 2:16020 doi: 10.1038/nrdp.2016.20 27158839 10.1038/nrdp.2016.20 PMC5453186

[3] Slimings C, Riley TV (2014) Antibiotics and hospital-acquired *Clostridium difficile* infection: Update of systematic review and meta-analysis. J Antimicrob Chemther 69:881–891. 10.1093/jac/dkt477 24324224

[4] Hensgens MPM, Goorhuis A, Dekkers OM, Kuijper EJ (2012) Time interval of increased risk for *Clostridium difficile* infection after exposure to antibiotics. J Antimicrob Chemther 67:742–748. 10.1093/jac/dkr508 22146873

[5] Marston HD, Dixon DM, Knisely JM, Palmore TN, Fauci AS (2016) Antimicrobial resistance. JAMA 316:1193–1204. doi: 10.1001/jama.2016.11764 27654605 10.1001/jama.2016.11764

[6] Tickler IA, Goering R V., Whitmore JD, Lynn ANW, Persing DH, Tenover FC (2014) Strain types and antimicrobial resistance patterns of *Clostridium difficile* isolates from the United States, 2011 to 2013. Antimicrob Agents Chemother 58:4214–4218. doi: 10.1128/AAC.02775-13 24752264 10.1128/AAC.02775-13 PMC4068552

[7] Dong D, Zhang L, Chen X, Jiang C, Yu B, Wang X, et al (2013) Antimicrobial susceptibility and resistance mechanisms of clinical *Clostridium difficile* from a Chinese tertiary hospital. Int J Antimicrob Agents 41:80–84. doi: 10.1016/j.ijantimicag.2012.08.011 23148985 10.1016/j.ijantimicag.2012.08.011

[8] Hecht DW, Galang MA, Sambol SP, Osmolski JR, Johnson S, Gerding DN (2007) In vitro activities of 15 antimicrobial agents against 110 toxigenic *Clostridium difficile* clinical isolates collected from 1983 to 2004. Antimicrob Agents Chemother 51:2716–2719. doi: 10.1128/AAC.01623-06 17517836 10.1128/AAC.01623-06 PMC1932509

[9] Elliott B, Reed R, Chang BJ, Riley TV (2009) Bacteremia with a large clostridial toxin-negative, binary toxin-positive strain of *Clostridium difficile*. Anaerobe 15:249–251. doi: 10.1016/j.anaerobe.2009.08.006 19723585 10.1016/j.anaerobe.2009.08.006

[10] Kuehne SA, Cartman ST, Minton NP (2011) Both, toxin A and toxin B, are important in *Clostridium difficile* infection. Gut Microbes 2:711–713. 10.4161/gmic.2.4.16109 PMC3260544 21804353

[11] Halstead DC, Abid J, Sloan L, Meza D, Ramsey-Walker D, Hata DJ (2016) A multi-laboratory comparison of two molecular methods for the detection of toxigenic *Clostridium difficile*. J Infect Dev Ctries 10:62–67. doi: 10.3855/jidc.6634 26829538 10.3855/jidc.6634

[12] Wilcox MH, Chalmers JD, Nord CE, Freeman J, Bouza E (2016) Role of cephalosporins in the era of *Clostridium difficile* infection. J Antimicrob Chemother 72:1–18. doi: 10.1093/jac/dkw385 27659735 10.1093/jac/dkw385 PMC5161048

[13] Wilkins TD, Lyerly DM (2003) *Clostridium difficile* Testing after 20 Years, Still Challenging. J Clin Microbiol 41:531–534. doi: 10.1128/JCM.41.2.531-534.2003 12574241 10.1128/JCM.41.2.531-534.2003 PMC149726

[14] Lopez-Urena D, Quesada-Gomez C, Miranda E, Fonseca M, Rodríguez-Cavallini E (2014) Spread of epidemic *Clostridium difficile* NAP1/027 in Latin America: Case reports in panama. J Med Microbiol 63:322–324. doi: 10.1099/jmm.0.066399-0 24287669 10.1099/jmm.0.066399-0

[15] Legaria MC, Lumelsky G, Rosetti S (2003) *Clostridium difficile*-associated diarrhea from a general hospital in Argentina. Anaerobe 9:113–116. doi: 10.1016/S1075-9964(03)00088-X 16887697

[16] Lopardo G, Morfin-Otero R, Moran-Vazquez II, Noriega F, Zambrano B, Luxemburger C, et al (2015) Epidemiology of *Clostridium difficile*: A hospital-based descriptive study in Argentina and Mexico. Braz J Infect Dis 19:8–14. doi: 10.1016/j.bjid.2014.07.004 25179510 10.1016/j.bjid.2014.07.004 PMC9425260

[17] Garcia C, Samalvides F, Vidal M, Gotuzzo E, Dupont HL (2007) Epidemiology of *Clostridium difficile*-associated diarrhea in a Peruvian tertiary care hospital. Am J Trop Med Hyg 77:802–805. 17984329

[18] Balassiano IT, Yates EA, Domingues RMCP, Ferreira EO (2012) *Clostridium difficile*: A problem of concern in developed countries and still a mystery in Latin America. J Med Microbiol 61:169–179. doi: 10.1099/jmm.0.037077-0 22116982 10.1099/jmm.0.037077-0

[19] Becerra MG, Ospina S, Atehortúa SL, Berbesi DY (2011) Factores de riesgo para la infección por *Clostridium difficile* [Risk factors for *Clostridium difficile* infection] Infectio 15:220–226.

[20] de Almeida Monteiro A, Pires RN, Persson S, Filho EMR, Pasqualotto AC (2014) A search for *Clostridium difficile* ribotypes 027 and 078 in Brazil. Braz J Infect Dis 18:672–674. doi: 10.1016/j.bjid.2014.08.004 25307680 10.1016/j.bjid.2014.08.004 PMC9425211

[21] Balassiano IT, Miranda KR, Boente RF, Pauer H, Oliveira ICM, Santos-Filho J, et al (2009) Characterization of *Clostridium difficile* strains isolated from immunosuppressed inpatients in a hospital in Rio de Janeiro, Brazil. Anaerobe 15:61–64. doi: 10.1016/j.anaerobe.2008.12.007 19154793 10.1016/j.anaerobe.2008.12.007

[22] Balassiano IT, Dos Santos-Filho J, Vital-Brazil JM, Nouér SA, Souza CRC, Brazier JS, et al (2011) Detection of cross-infection associated to a Brazilian PCR-ribotype of *Clostridium difficile* in a university hospital in Rio de Janeiro, Brazil. Antonie van Leeuwenhoek, Int J General Mol Microbiol 99:249–255. 10.1007/s10482-010-9483-8 20623188

[23] Oñate-Gutierrez J, Villegas M, Correa A (2016) Prevalencia y factores relacionados con la infección por *Clostridium difficile* en un centro hospitalario de alta complejidad en Cali (Colombia) [Prevalence and factors related to *Clostridium difficile* infection in a highly complex hospital center in Cali (Colombia)]. Infectio 21:9–14

[24] Surawicz CM, Brandt LJ, Binion DG, Ananthakrishnan AN, Curry SR, Gilligan PH, et al (2013). Guidelines for diagnosis, treatment, and prevention of *Clostridium difficile* infections. Am J Gastroenterol 108:478–498. doi: 10.1038/ajg.2013.4 23439232 10.1038/ajg.2013.4

[25] Foster NF, Riley TV (2012) Improved recovery of *Clostridium difficile* spores with the incorporation of synthetic taurocholate in cycloserine-cefoxitin-fructose agar (CCFA). Pathology 44:354–356. doi: 10.1097/PAT.0b013e328353a235 22531346 10.1097/PAT.0b013e328353a235

[26] Clinical and Laboratory Standards Institute (2013) M100-S23. Performance standards for antimicrobial susceptibility testing: 23th informational supplement, 23th ed Clinical and Laboratory Standards Institute, Wayne, Pennsylvania http://reflab.yums.ac.ir/uploads/clsi_m100-s23-2013.pdf

[27] Hernández-rocha C, Barra-carrasco J, Álvarez-lobos M (2013) Prospective comparison of a commercial multiplex real-time polymerase chain reaction and an enzyme immunoassay with toxigenic culture in the diagnosis of *Clostridium difficile*–associated infections. Diagn Microbiol Infect Dis 75:361–365. doi: 10.1016/j.diagmicrobio.2012.12.010 23415540 10.1016/j.diagmicrobio.2012.12.010

[28] Kato N, Ou C-Y, Kato H, Bartley SL, Brown VK, Dowell VR, et al (1991) Identification of Toxigenic *Clostridium dijficile* by the Polymerase Chain Reaction. J Clin Microbiol 29:33–37. 1993763 10.1128/jcm.29.1.33-37.1991 PMC269697

[29] Terhes G, Urbán E, Sóki J, Hamid KA, Nagy E (2004) Community-acquired *Clostridium difficile* diarrhea caused by binary toxin, toxin A, and toxin B gene-positive isolates in Hungary. J Clin Microbiol 42:4316–4318. doi: 10.1128/JCM.42.9.4316-4318.2004 15365032 10.1128/JCM.42.9.4316-4318.2004 PMC516352

[30] Chia JH, Lai HC, Su LH, Kuo AJ, Wu TL (2013) Molecular Epidemiology of *Clostridium difficile* at a Medical Center in Taiwan: Persistence of Genetically Clustering of A-B+ Isolates and Increase of A+B+ Isolates. PLoS One 8:e75471 doi: 10.1371/journal.pone.0075471 24116048 10.1371/journal.pone.0075471 PMC3792110

[31] Fawley WN, Knetsch CW, MacCannell DR, Harmanus C, Du T, Mulvey MR, et al (2015) Development and validation of an internationally-standardized, high-resolution capillary gel-based electrophoresis PCR-ribotyping protocol for *Clostridium difficile*. PLoS One 10:e0118150 doi: 10.1371/journal.pone.0118150 25679978 10.1371/journal.pone.0118150 PMC4332677

[32] Crabtree T, Aitchison D, Meyers BF, Tymkew H, Smith JR, Guthrie TJ, et al (2007) *Clostridium difficile* in cardiac surgery: risk factors and impact on postoperative outcome. Ann Thorac Surg 83:1396–1402. doi: 10.1016/j.athoracsur.2006.10.067 17383346 10.1016/j.athoracsur.2006.10.067

[33] Fehér C, Mensa J (2016) A Comparison of Current Guidelines of Five International Societies on *Clostridium difficile* Infection Management. Infect Dis Ther 5:207–230. doi: 10.1007/s40121-016-0122-1 27470257 10.1007/s40121-016-0122-1 PMC5019978

[34] Foster NF, Collins DA, Ditchburn SL, Duncan CN, van Schalkwyk JW, Golledge CL, et al (2014) Epidemiology of *Clostridium difficile* infection in two tertiary-care hospitals in Perth, Western Australia: A cross-sectional study. New Microbes New Infect 2:64–71. doi: 10.1002/nmi2.43 25356346 10.1002/nmi2.43 PMC4184660

[35] Karanika S, Paudel S, Zervou FN, Grigoras C, Zacharioudakis IM, Mylonakis E (2016) Prevalence and clinical outcomes of *Clostridium difficile* infection in the intensive care unit: A systematic review and meta-analysis. Open Forum Infect Dis 3:ofv186. 10.1093/ofid/ofv186 PMC4716350 26788544

[36] Wijarnpreecha K, Sornprom S, Thongprayoon C, Phatharacharukul P, Cheungpasitporn W, Nakkala K (2016) The risk of *Clostridium difficile* associated diarrhea in nasogastric tube insertion: A systematic review and meta-analysis. Dig Liver Dis 48:468–472. doi: 10.1016/j.dld.2016.01.012 26905926 10.1016/j.dld.2016.01.012

[37] Leibovitz A, Yarovoy A, Sharshar N, Buckman Z, Mizrahi EH, Lubart E (2016) *Clostridium difficile*-associated disease: A primary clinical evaluation of elderly patients in a geriatric hospital. A J Infect Control 44:1158–1160. 10.1016/j.ajic.2016.03.069 27375063

[38] Dudukgian H, Sie E, Gonzalez-Ruiz C, Etzioni DA, Kaiser AM (2010) *C*. *difficile* colitis-predictors of fatal outcome. J Gastrointes Surg 14:315–322. 10.1007/s11605-009-1093-2 19937192

[39] Bloomfield MG, Sherwin JC, Gkrania-Klotsas E (2012) Risk factors for mortality in *Clostridium difficile* infection in the general hospital population: A systematic review. J Hosp Infect 82:1–12. doi: 10.1016/j.jhin.2012.05.008 22727824 10.1016/j.jhin.2012.05.008

[40] Kim HB, Wang Y, Sun X (2016) A detrimental role of immunosuppressive drug, dexamethasone, during *Clostridium difficile* infection in association with a gastrointestinal microbial shift. J Microbiol Biotechnol 26:567–571. doi: 10.4014/jmb.1512.12017 26809802 10.4014/jmb.1512.12017 PMC4832933

[41] Tleyjeh IM, Bin Abdulhak AA, Riaz M, Alasmari FA, Garbati MA, AlGhamdi M, et al (2012) Association between Proton Pump Inhibitor Therapy and *Clostridium difficile* Infection: A Contemporary Systematic Review and Meta-Analysis. PLoS One 7:e50836 doi: 10.1371/journal.pone.0050836 23236397 10.1371/journal.pone.0050836 PMC3517572

[42] Dial S, Alrasadi K, Manoukin C, Huang A, Menzies D (2004) Risk of *Clostridium difficile* diaarhea among hospital inpatients prescribed proton pump inhibitors: Cohort and case-control studies. CMAJ 171:33–38. doi: 10.1503/cmaj.1040876 15238493 10.1503/cmaj.1040876 PMC437681

[43] Faleck DM, Salmasian H, Furuya EY, Larson EL, Abrams JA, Freedberg DE (2016) Proton pump inhibitors do not affect risk for *Clostridium difficile* infection in the intensive care unit. Am J Gastroenterol 111:1641–1648. doi: 10.1038/ajg.2016.343 27575714 10.1038/ajg.2016.343 PMC5096970

[44] Magill SS, Edwards JR, Bamberg W, Beldavs ZG, Dumyati G, Kainer MA, et al (2014) Multistate Point-Prevalence Survey of Health Care—Associated Infections. N Engl J Med 370:1198–1208. doi: 10.1056/NEJMoa1306801 24670166 10.1056/NEJMoa1306801 PMC4648343

[45] Ashwin H, Longshaw C, Ashwin H, Davies KA, Davis GL, Lee F, et al. (2014) Optimised diagnosis of Clostridium difficile infection; is there still room for improvement? Results of a European point prevalence study of C. difficile infection (EUCLID) Poster presented at 24th European Congress of Clinical Microbiology and Infectious Diseases (ECCMID).

[46] Eastwood K, Else P, Charlett A, Wilcox M (2009) Comparison of nine commercially available *Clostridium difficile* toxin detection assays, a real-time PCR assay for *C*. *difficile* tcdB, and a glutamate dehydrogenase detection assay to cytotoxin testing and cytotoxigenic culture methods. J Clin Microbiol 47:3211–3217. doi: 10.1128/JCM.01082-09 19710274 10.1128/JCM.01082-09 PMC2756932

[47] Sunkesula VCK, Kundrapu S, Muganda C, Sethi AK, Donskey CJ (2013) Does empirical *Clostridium difficile* infection (CDI) therapy result in false-negative CDI diagnostic test results? Clin Infect Dis 57:494–500. doi: 10.1093/cid/cit286 23645849 10.1093/cid/cit286

[48] Freeman J, Bauer MP, Baines SD, Corver J, Fawley WN, Goorhuis B, et al (2010) The changing epidemiology of *Clostridium difficile* infections. Clin Microbiol Rev 23:529–549. doi: 10.1128/CMR.00082-09 20610822 10.1128/CMR.00082-09 PMC2901659

[49] Freeman J, Vernon J, Morris K, Nicholson S, Todhunter S, Longshaw C, et al (2015) Pan-European longitudinal surveillance of antibiotic resistance among prevalent *Clostridium difficile* ribotypes. Clin Microbiol Infect 21:248.e9–248.e16. 10.1016/j.cmi.2014.09.017 25701178

[50] Krutova M, Nyc O, Matejkova J, Allerberger F, Wilcox MH, Kuijper EJ. (2016) Molecular characterisation of Czech *Clostridium difficile* isolates collected in 2013–2015. Int J Med Microbiol 306:479–485. doi: 10.1016/j.ijmm.2016.07.003 27519407 10.1016/j.ijmm.2016.07.003

